# Mechanistic insights into HapC: A key enzyme in prodiginine biosynthesis in *Hahella chejuensis*

**DOI:** 10.1111/febs.70518

**Published:** 2026-03-30

**Authors:** Yueh‐Te Chan, Aden Dhana Rizkita, Hsin‐Ju Hsieh, Shu‐Fang Hsu, Tony Eight Lin, Wei‐Jan Huang, Kai‐Cheng Hsu, Sheila Nathan, Peiching Chang, Andrew H.‐J. Wang, Cheng‐Chung Lee

**Affiliations:** ^1^ The Ph.D. Program for Translational Medicine, College of Medical Science and Technology Taipei Medical University Taiwan; ^2^ School of Pharmacy, College of Pharmacy Taipei Medical University Taiwan; ^3^ Department of Water System Development and Application Research, Division of Water Technology Industrial Technology Research Institute Hsinchu Taiwan; ^4^ Material and Chemical Research Laboratories Industrial Technology Research Institute Hsinchu Taiwan; ^5^ Graduate Institute of Cancer Biology and Drug Discovery, College of Medical Science and Technology Taipei Medical University Taiwan; ^6^ Ph.D. Program for Cancer Molecular Biology and Drug Discovery, College of Medical Science and Technology Taipei Medical University Taiwan; ^7^ Ph.D. Program in Drug Discovery and Development Industry, College of Pharmacy Taipei Medical University Taiwan; ^8^ Faculty of Science and Technology, Department of Biological Sciences and Biotechnology Universiti Kebangsaan Malaysia Bangi Selangor Malaysia; ^9^ International Ph.D. Program for Translational Science, College of Medical Science and Technology Taipei Medical University Taiwan

**Keywords:** biocontrol agents, drug discovery, *Hahella chejuensis*, prodiginine, prodigiosin

## Abstract

Research to understand the enzymatic machinery underlying the biosynthesis of red prodigiosin, a potent secondary metabolite with significant biological activity, led to the identification of HapC, an enzyme from the marine bacterium *Hahella chejuensis*. In this study, we demonstrated the catalytic capability of HapC in synthesizing a diverse array of short‐chain prodiginines through the condensation of various 2‐methyl‐3‐amylpyrrole (MAP) analogs with 4‐methoxy‐2,2′‐bipyrrole‐5‐carboxaldehyde (MBC). Six prodiginines, all non‐native to HapC, were enzymatically synthesized *in vitro*, including two analogs, 3,4‐dimethyl‐6‐methoxyprodiginine and 2‐ethyl‐6‐methoxyprodiginine, that have not been successfully produced by any prodigiosin‐forming enzyme to date, demonstrating the subtle differences in the products formed by HapC. Constructing a structural model of HapC and performing ligand docking simulations with representative ligands corresponding to different reaction steps, including an ATP analog, a phosphorylated MBC, and a prodiginine product, revealed catalytic residues in the two active sites of HapC. To extend from local catalytic interactions to the global mechanistic level, a comparative structural analysis with the homologous rifampin phosphotransferase from *Listeria monocytogenes* was applied to infer domain dynamics in HapC. A well‐defined three‐phase catalytic cycle was delineated, highlighting the role of domain rearrangements and phosphotransfer mediated by the conserved histidine residue H859. These findings enhance our understanding of HapC, with the potential to diversify prodiginine libraries and guide rational drug design.

## Graphical abstract

HapC catalyzes prodiginine biosynthesis through coordinated movements of its ATP‐binding, substrate‐binding, and catalytic‐swivel domains. The enzyme shows tolerance toward short‐chain MAP analogs, and docking analyses reveal key residues and structural features underlying its substrate flexibility.

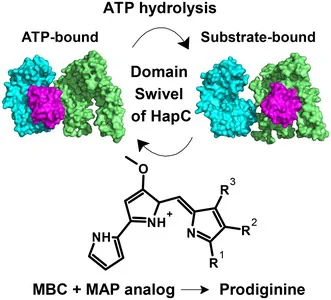

**Abbreviations**
ABD: ATP‐binding domain
AMPPNP: adenylyl‐imidodiphosphate
CSD: catalytic swivel domain
*H. chejuensis*
: *Hahella chejuensis*
MAP: 2‐methyl‐3‐amylpyrrole
MBC: 4‐methoxy‐2,2′‐bipyrrole‐5‐carboxaldehyde
MBC‐P: phosphorylated MBC
RPH: rifampin phosphotransferase
SBD: substrate‐binding domain

## Introduction

**Fig. 1:**
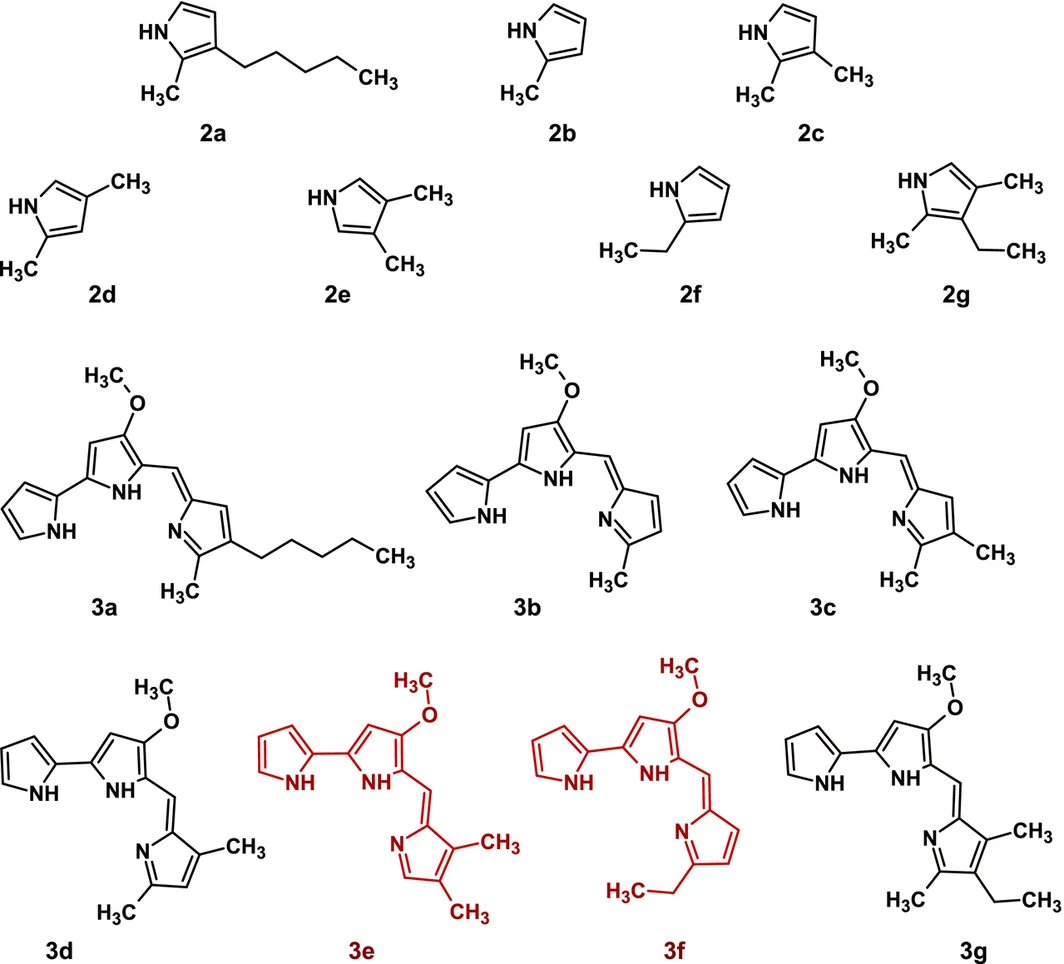
Structural overview of MAP (2a) and MAP analogs (2b–2g) and their corresponding prodiginine products (3a–3g). New prodiginines (red) refer to enzymatic *in vitro* derivatives not previously reported among known synthetic analogs.

Microbial secondary metabolites form a diverse and chemically rich reservoir with significant therapeutic potential [1, 2, 3]. Among these compounds, prodigiosin stands out not only for its striking red color but also for its array of bioactive properties [4, 5]. Originally identified in terrestrial *Serratia* species, prodigiosin is the parent compound of the prodiginine family, characterized by a distinctive tripyrrole structure [6] (3a in Fig. 1). Previous studies underscore the significance of prodigiosin in agriculture, highlighting its efficacy against plant pathogens, fungi, and nematodes [5]. In human medicine, prodigiosin has demonstrated antibacterial [4, 7, 8], anticancer [9, 10], and immunosuppressive bioactivity [6, 11], indicating its therapeutic potential.

The biological activities of prodigiosin are closely linked to its interactions with biomolecules. It binds DNA and can trigger apoptosis, potentially via p53 modulation [7, 12, 13, 14]. Prodigiosin forms bioactive metal complexes [15], with Pt(II) in some cases adopting terpyridine‐like coordination [16, 17]. It also engages proteins, which may enhance its solubility and stability [14, 18]. Among these, studies of prodigiosin‐DNA binding are the most extensive. The tripyrrole scaffold of prodigiosin intercalates into the DNA minor groove via hydrogen bonding and hydrophobic contacts [7, 19, 20]. The DNA‐binding affinity also depends on side chain length, with short‐chain prodiginines exhibiting higher affinity than longer‐chain counterparts [21].

The biosynthesis of prodigiosin has been studied since the 1970s [22, 23, 24, 25]. Two separate pathways generate the late‐stage intermediates 4‐methoxy‐2,2′‐bipyrrole‐5‐carboxaldehyde (MBC) and 2‐methyl‐3‐amylpyrrole (MAP) (2a in Fig. 1), which undergo final‐step condensation to form prodigiosin. Early studies on prodigiosin primarily focused on *Serratia marcescens (S. marcescens)*. Interest later expanded to the marine bacterium *Hahella chejuensis (H. chejuensis)*, which produces prodigiosin as a key metabolite with significant algicidal activity [4, 26, 27]. The prodigiosin biosynthesis gene clusters in *H. chejuensis* and *S. marcescens*, known as the *hap* and *pig* clusters, respectively [26, 28, 29], exhibit significant structural conservation (Fig. S1). The genes *hapB/pigB*, *hapD/pigD*, and *hapE/pigE* are involved in the biosynthesis of MAP, whereas *hapA/pigA* and *hapF–N/pigF–N* are associated with the biosynthesis of MBC. The enzymes HapC and PigC, encoded by the *hap*C and *pigC* genes, respectively [9, 26, 30], catalyze the final step in prodigiosin synthesis by facilitating the condensation of MBC and MAP [7, 31]. Notably, HapC is less studied than PigC but has been reported to exhibit higher catalytic activity [9]. In addition, protein structural studies have illuminated the pathway, with crystal structures available for several upstream enzymes [29, 32, 33, 34]. However, both HapC and PigC remain structurally uncharacterized, and a clear framework linking their architecture to catalytic function is still lacking.

In this study, we evaluated HapC *in vitro* with substrates that systematically vary the terminal pyrrole unit, derived from short‐chain MAP analogs (2b–2g in Fig. 1). The successful synthesis of six non‐native prodiginines (3b–3g) highlights the flexible substrate recognition of HapC. To investigate its catalytic mechanism, a structural model of HapC was constructed. Ligand docking simulations identified key residues within the active sites. Combined with comparative structural analyses of HapC against the homologous rifampin phosphotransferase (RPH) [35], a mechanistic model of HapC is proposed. These results advance our understanding of HapC catalysis and open promising avenues for prodiginine‐based drug design.

## Results

### Structural analysis and domain architecture of HapC

**Fig. 2:**
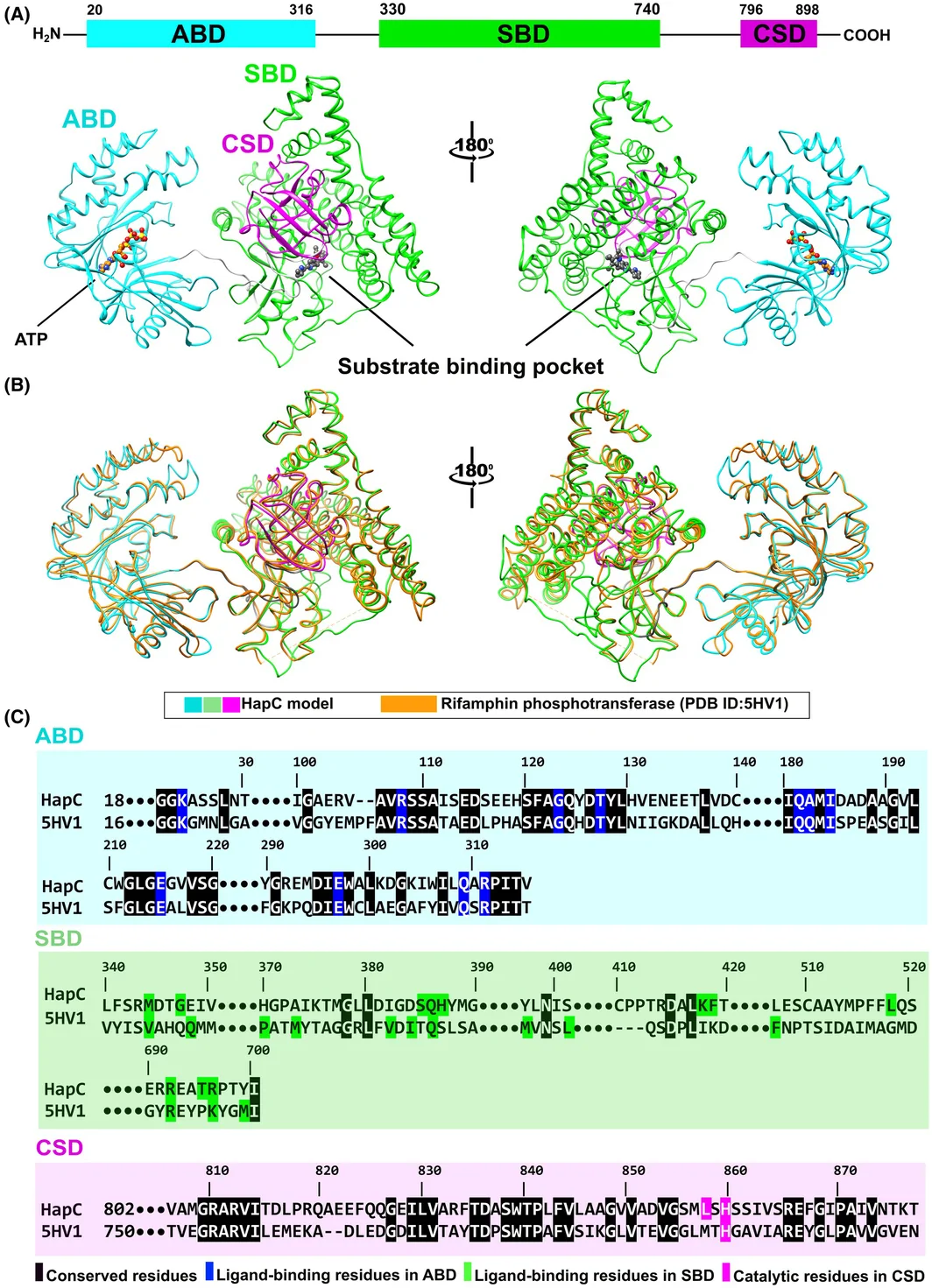
Predictive structure model of HapC from *Hahella chejuensis*. (A) Schematic representation of HapC domains: the ABD (residues 20–316), SBD (residues 330–740), and CSD (residues 796–898) are shown in cyan, green, and magenta, respectively. The predictive 3D structure of HapC and 180° rotation view are shown in ribbon representation. The binding ligand AMPPNP and the product prodiginine (3g) are depicted as orange and dark gray sticks, respectively. (B) Structural comparison of HapC and rifampin phosphotransferase (RPH) (PDB ID: 5HV1). HapC is shown as a multicolored ribbon corresponding to its domains, whereas RPH is depicted as an orange ribbon. (C) Sequence alignment of the ABD, SBD, and CSD of HapC and RPH. Conserved residues are shaded in black. Residues involved in hydrogen bonding with ligand in ABD are marked in blue. In the SBD, substrate‐binding residues are indicated in green. The catalytic histidine involved in phosphate transfer is highlighted in magenta. The HapC homology model was constructed using the SWISS‐MODEL server. Structural analyses and graphical representations were generated using UCSF Chimera. Sequence alignment was performed using ClustalW and formatted using Boxshade.

Since no crystal structure of HapC is available, a sequence‐based search was conducted to identify suitable templates for homology modeling. PDB: 5HV1, representing RPH from *Listeria monocytogenes*, showed the highest sequence identity (27.3%) and near‐complete coverage (904/908 residues) with HapC. It was selected as the template for generating the 5HV1‐based HapC model. The stereochemical quality of the model was evaluated using the SWISS‐MODEL Expasy validation pipeline (Fig. S2). The model achieved a MolProbity score of 1.98 with a clash score of 2.34, indicating good stereochemical quality. Ramachandran analysis showed 93.63% of residues in favored or allowed regions, consistent with a reliable model. The HapC model clearly reveals a three‐domain architecture (Fig. 2A), which includes an ATP‐binding domain (ABD) spanning residues 20–316; a substrate‐binding domain (SBD) spanning residues 330–740, corresponding to the ligand‐binding region; and a catalytic swivel domain (CSD) in the C‐terminal region (residues 796–898) [35]. Superimposing the 5HV1‐based HapC onto the template RPH yielded a root mean square deviation (RMSD) of 0.75 Å between 593 pruned atom pairs (Fig. 2B). Alignment of HapC domains with the RPH template (5HV1) revealed domain‐specific sequence identities: 34.5% for the N‐terminal ABD, 21.6% for the SBD, which is the lowest among the three, and 37.6% for the C‐terminal CSD, representing the highest identity among the three domains (Fig. 2C).

### *In vitro* characterization of pyrrole acceptance by HapC

**Fig. 3:**
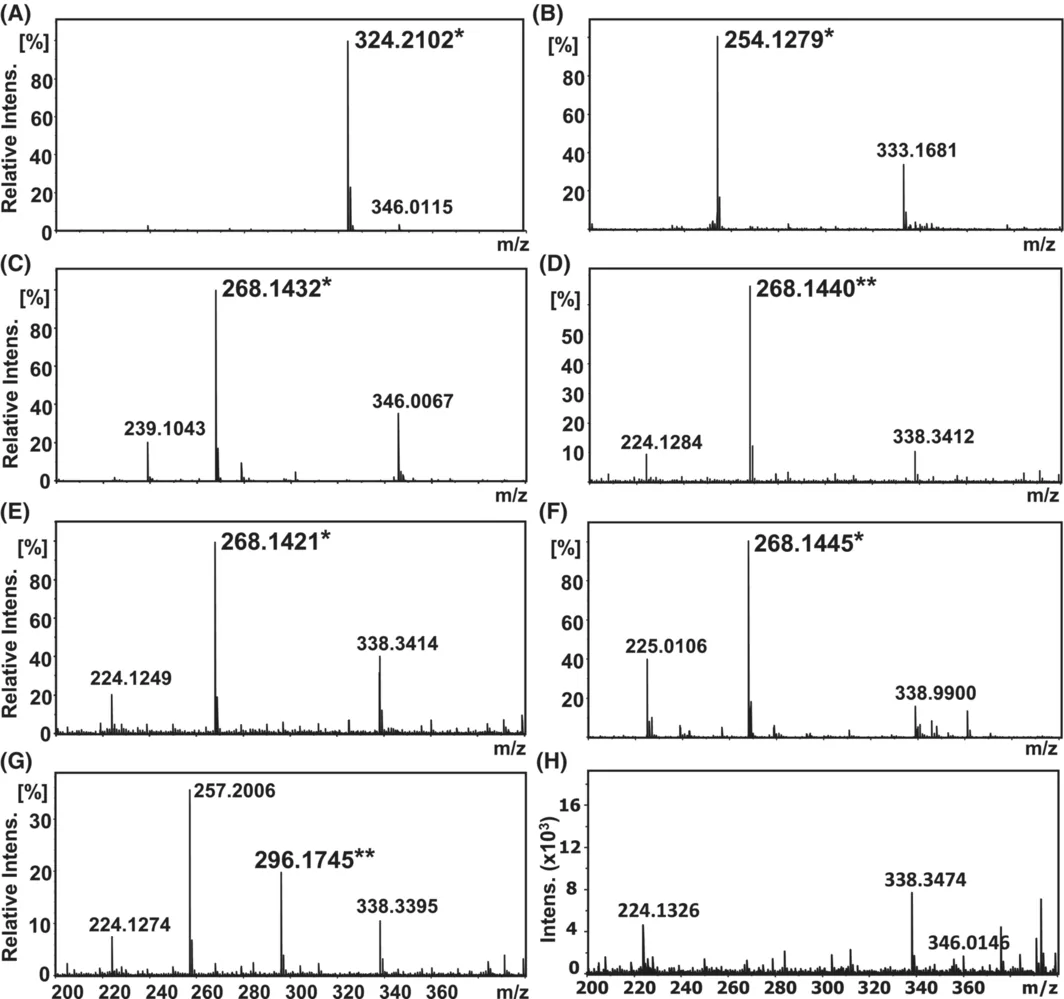
LC–MS high‐resolution mass spectra of enzymatic products generated by HapC. Panels (A–G) correspond to products 3a–3g, respectively. The corresponding extracted ion chromatograms are shown in Fig. S3. Panel (H) shows the blank check confirming the absence of product‐related ions. Signals at *m/z* 224, 338, and 346 are attributed to background ions. The calculated [M + H]^+^ peaks are marked with asterisks (*). In panels (D) and (G), the internal calibrant signal was stronger than that of the target ion (**); therefore, relative intensities were normalized to the calibrant. In panel (B), the ion observed at *m/z* 333.1681 is likely attributable to a pyridine adduct [57], [M + H + C_5_H_5_N]^+^. Panel (C) shows a fragment ion at *m/z* 239.10, which was also detected as an early‐eluting peak in compounds 3d and 3e (Fig. S3D,E). The absence of this signal in the blank check suggests that it originates from in‐source fragmentation involving a neutral loss of approximately 29 Da (consistent with CHO) [58]. For panel (G) ([M + H]^+^ = 296.1757), the fragment at *m/z* 257.2 is proposed to arise from *β*‐cleavage and allylic rearrangement at the substituted pyrrole terminus [59], generating a resonance‐stabilized dipyrrolylmethenium‐type cation (*m/z* 257.15). LC–MS data were acquired using the Bruker Maxis Impact system as described in the Materials and Methods.

To assess the catalytic versatility of HapC, a series of short‐chain MAP analogs featuring structural variations at the *R* ^1^, *R* ^2^, and *R* ^3^ positions were tested *in vitro* (Schema 1; Fig. 1). Prodigiosin (3a), generated from MAP (2a), was used as a positive control. All synthesized compounds (3a–3g) exhibited visible absorption maxima between 521 and 539 nm (Table 1), consistent with the presence of an extended conjugated system characteristic of prodiginine‐type scaffolds [36]. LC–MS analysis of each reaction revealed dominant [M + H]^+^ ions consistent with the formation of the expected products (Table 1). The mass spectra of compounds 3a–3g are shown in Fig. 3A–G. Although background signals were present in the blank control (Fig. 3H), no peaks corresponding to the expected products were detected. The extracted ion chromatograms are presented in Fig. S3A–G, with the background control shown in Fig. S3H. Structural characterization was performed by ^1^H NMR spectroscopy, and the resulting spectra showed chemical shifts and coupling patterns consistent with the proposed molecular structures (Data S1; Figs S4–S10). To the best of our knowledge, two of the products, 3,4‐dimethyl‐6‐methoxyprodiginine (3e) and 2‐ethyl‐6‐methoxyprodiginine (3f), have not been successfully produced by other prodiginine enzymes. Notably, 3f was tested with PigC but showed no activity [37], highlighting the potential of this approach for generating new compounds. The other four products (3b, 3c, 3d, and 3g) correspond to previously known prodiginines [37, 38], now demonstrated to be accessible through HapC catalysis.

**Schema 1:**
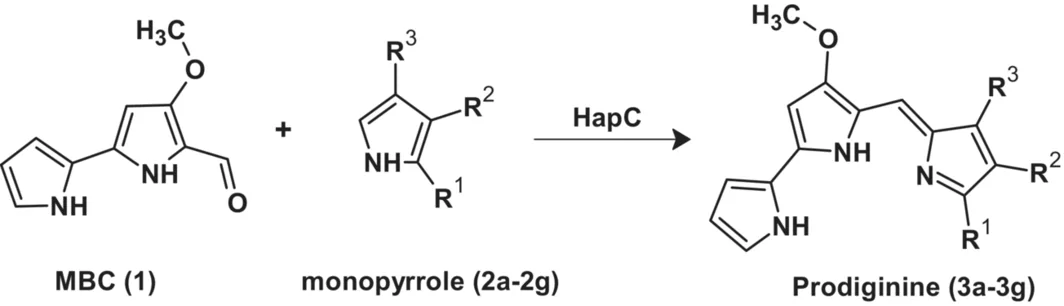
Enzymatic biosynthesis of prodiginines (3a–3g in Fig. 1) via condensation of MBC (1) with monopyrroles (2a–2g) catalyzed by HapC. *R* ^1^, *R* ^2^, and *R* ^3^ indicate variable substituents on the monopyrrole ring.

**Table 1:** Prodiginines (3a–3g) synthesized by HapC from MBC and monopyrroles (2a–2g). Each entry corresponds to a distinct reaction using MAP (2a) or a specific MAP analog (2b–2g). Substituent positions *R* ^1^, *R* ^2^, and *R* ^3^ define the structural variation on the monopyrrole ring. The product mass (g·mol^−1^), as determined by LC–MS, and the absorption maximum (*λ*) are listed.

No.	Monopyrrole substrate	*R* ^1^	*R* ^2^	*R* ^3^	Product	Product mass	Product abs. *λ* (nm)
1	2a	methyl	n‐pentyl	H	3a	323.210	539
2	2b	methyl	H	H	3b	253.128	523
3	2c	methyl	methyl	H	3c	267.143	533
4	2d	methyl	H	methyl	3d	267.144	527
5	2e	H	methyl	methyl	3e	267.142	522
6	2f	ethyl	H	H	3f	267.145	521
7	2g	methyl	ethyl	methyl	3g	295.175	533

### Docking simulation of the ATP‐binding domain in HapC

**Fig. 4:**
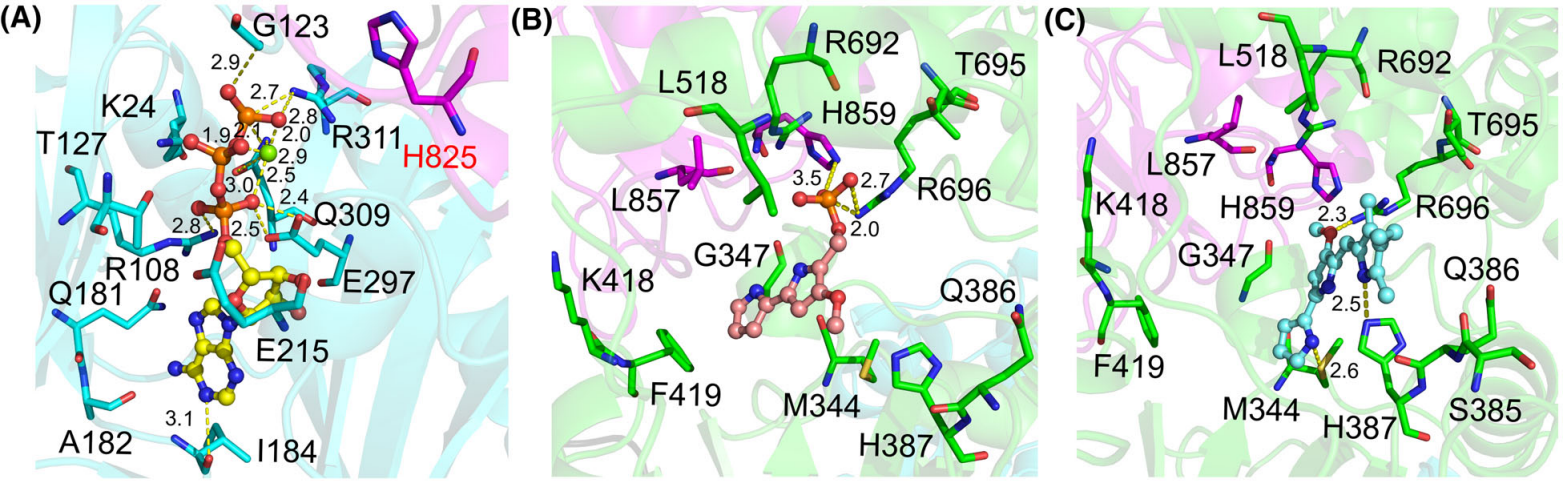
Ligand docking simulations of HapC. (A) Docking of ATP in the ABD. ATP is rendered as yellow spheres and sticks, and the Mg^2+^ ion is shown as a green sphere. The phosphate groups are colored orange. Hydrogen‐bonding interactions between ATP and surrounding residues are indicated by yellow dashed lines, with interatomic distances labeled in angstroms (Å). The red‐colored histidine (H825) is derived from a superimposed structure (PDB ID: 5HV2) to illustrate the approximate spatial position of the corresponding catalytic residue H859 in HapC during the reaction. (B) Docking of the phosphorylated intermediate MBC (MBC‐P) in the SBD. MBC‐P is shown in salmon. (C) Docking of the final product, 3‐ethyl‐2,4‐dimethyl‐6‐methoxyprodiginine (3g), in the SBD. The product is depicted in light blue. Residues in the SBD and CSD are represented as green and magenta sticks, respectively. Hydrogen‐bonding interactions with active‐site residues are indicated by yellow dashed lines, with distances labeled in Å. Docking simulations were performed using the CDOCKER module in Discovery Studio. Molecular graphics were rendered using UCSF Chimera.

Further analysis of the ABD in HapC used docking to identify key ATP‐binding residues. The crystal structure of RPH with AMPPNP, a nonhydrolyzable analog of ATP, bound at the ABD (PDB: 5HV1) was used as a reference. Docking of ATP with the HapC model demonstrated that ATP binds favorably within the ABD. Structural comparison revealed that the ABD of HapC is conserved with that of RPH. These include conserved residues (HapC/RPH) K24/K22, R108/R117, G123/G132, T127/T136, Q181/Q183, I184/I186, E215/E217, E297/E297, Q309/Q309, and R311/R311, which are critical for ATP binding (Fig. S11). The docking simulations indicated a stable and energetically favorable interaction between the substrate and the active‐site residues of HapC, with the adenine ring of ATP establishing hydrophobic interactions with and I184. The ABD facilitates the tight binding of ATP within the cleft through several conserved residues and an Mg^2+^ ion (Fig. 4A). Specifically, the α‐phosphate of ATP forms hydrogen bonds with E297 and R108, while the γ‐phosphate establishes hydrogen bonds with G123 and R311. In addition, the α‐, β‐, and γ‐phosphates are all coordinated with Q309 through the Mg^2+^ ion. The ATP molecule is further stabilized by hydrophobic interactions contributed by residues E215, A182, Q181, T127, and K24.

### Docking analysis of the SBD binding pocket

The ligand‐binding positions within the SBD of HapC were initially explored by docking the phosphorylated MBC (MBC‐P) into the active site (Fig. 4B), revealing interactions with residues G347, H387, K418, F419, L518, T695, R696, L857, and H859. Among these, H859 and L857 originate from the CSD, while the others belong to the SBD. Specifically, the guanidinium group of R696 and the imidazole of H859 form hydrogen bonds with the terminal phosphate oxygen of MBC‐P (Fig. 4B), with bond lengths of 2.6 Å and 3.5 Å, respectively.

In the context of product docking, 3‐ethyl‐2,4‐dimethyl‐6‐methoxyprodiginine (3g), which carries substituents at all three side chain positions (*R* ^1^, *R* ^2^, and *R* ^3^) of the pyrrole ring, was selected for docking into the active site of the SBD to enable more comprehensive structural inference [Correction added on 07 April 2026 after first online publication: The compound abbreviation “1g” has been replaced with “3g.”] The interacting network largely mirrors that of the MBC‐P complex but extends to include additional contacts with Q386, S385, and M344 (Fig. 4C). Moreover, hydrogen bonds are formed with H387 (2.5 Å), M344 (2.6 Å), and R696 (2.3 Å), concentrated around the MBC‐derived moiety of the prodiginine product.

## Discussion

### Structure‐guided annotation of functional residues in HapC

Among the homologs of HapC, PigC has been studied for its role in the synthesis of diverse prodiginines. For example, PigC from *Pseudoalteromonas* strains is involved in the biosynthesis of prodigiosin, cycloprodigiosin, and 2‐(p‐hydroxybenzyl)prodigiosin [6]. In another study, PigC cloned from *S. marcescens* demonstrated the ability to accommodate analogs with diverse alkyl chains at the *R* ^1^ and *R* ^2^ positions of the pyrrole ring (the *R*‐group positions are defined in Schema 1) [38]. With regard to HapC, side chain modifications at the *R* ^2^ position with longer alkyl groups, such as hexyl and octyl, are moderately tolerated. It has also been reported that substitution of the pyrrole nitrogen with sulfur or oxygen completely abolishes enzymatic activity [9]. In this study, a series of short‐chain MAP analogs with side chain modifications at positions *R* ^1^, *R* ^2^, and *R* ^3^ were employed (Table 1; Schema 1). In terms of molecular interactions, shorter side chains at specific positions may reduce steric hindrance and improve pharmacokinetic properties in medical applications [21]. Moreover, metal ions such as Cu^2+^ and Zn^2+^ can coordinate with prodigiosin, mediating interactions with bovine serum albumin [14]. Given the known metal coordination modes of prodigiosin, the side chain positions at *R* ^1^, *R* ^2^, and *R* ^3^ are less likely to interfere with metal binding, highlighting pyrrole modifications as a strategy to optimize prodiginine therapeutics.

In the modeling of HapC using the RPH structure as a template, conserved catalytic residues near the active site of the SBD are observed, including H859/H825, R692/R666 (HapC/RPH) (Fig. 2C). These residues are proposed to facilitate catalysis through catalytic base activation and ligand stabilization [35]. Histidine and arginine residues are frequently found near active sites of enzymes that interact with phosphate‐containing ligands [39, 40], consistent with our findings. Notably, in the docking model of MBC‐P in HapC, we observed dual engagement of R696 and H859 with the phosphate group, suggesting a cooperative stabilization (Fig. 4B). R696 neutralizes the negative charge of the phosphate and anchors the ligand in the cleft, whereas H859, situated near the catalytic site, not only stabilizes the phosphate but also serves as the phosphate donor during transfer. This dual recognition underscores the importance of both electrostatic and catalytic contributions. The finding is consistent with our mechanistic expectation that phosphorylation of MBC is catalyzed by H859‐P, the phosphorylated histidine from the CSD, leading to the formation of MBC‐P. R696 in the SBD forms hydrogen bonds with the phosphate group and the pyrrolic ring of MBC‐P, facilitating catalysis during the phosphate transfer.

To infer more functional features of the SBD in HapC, we referred to previous PigC models generated using the YASARA Structure suite [41, 42]. Within the SBD, conserved residues were identified between HapC and PigC, including (HapC/PigC) T346/T329, G347/G330, V350/V333, T351/T334, F620/F603, R691/R674, A694/A677, T695/T678, and P697/P680 (Fig. S12). Among these, G347 and T695 align with the HapC docking results, and together these residues delineate the substrate‐binding cavity. V350/V333 and T351/T334, positioned at the pocket entrance, likely serve as gatekeeper residues as suggested for PigC [41]. In HapC, V350 likely governs the entry orientation of MBC. MBC then traverses the tunnel to reach the inner region of the active site. The bipyrrole moiety of MBC is further stabilized by hydrophobic interactions with G347, H387, K418, F419, L518, T695, and L857 (Fig. 4B). L857 accompanies the catalytic residue H859, both originating from the CSD. While H859 engages directly in catalysis, L857 provides hydrophobic steric support and positional stabilization of the substrate. In the product docking model, the bipyrrole moiety derived from MBC remains anchored within the same cavity and forms tighter interactions with surrounding residues (Fig. 4C), including H387 (2.5 Å), M344 (2.6 Å), and R696 (2.3 Å), reflecting enhanced stabilization of the prodiginine scaffold. At the distal end, the monopyrrole moiety is surrounded by ample space, permitting accommodation of various substituents at *R* ^1^, *R* ^2^, and *R* ^3^, consistent with the observed substrate flexibility of HapC. We suggest that MBC binding is the primary determinant of substrate positioning, while the MAP analog serves as a reaction partner. These findings clarify key aspects of the catalytic mechanism of HapC at the active‐site level.

### Comparative structural analysis of HapC across different models

**Fig. 5:**
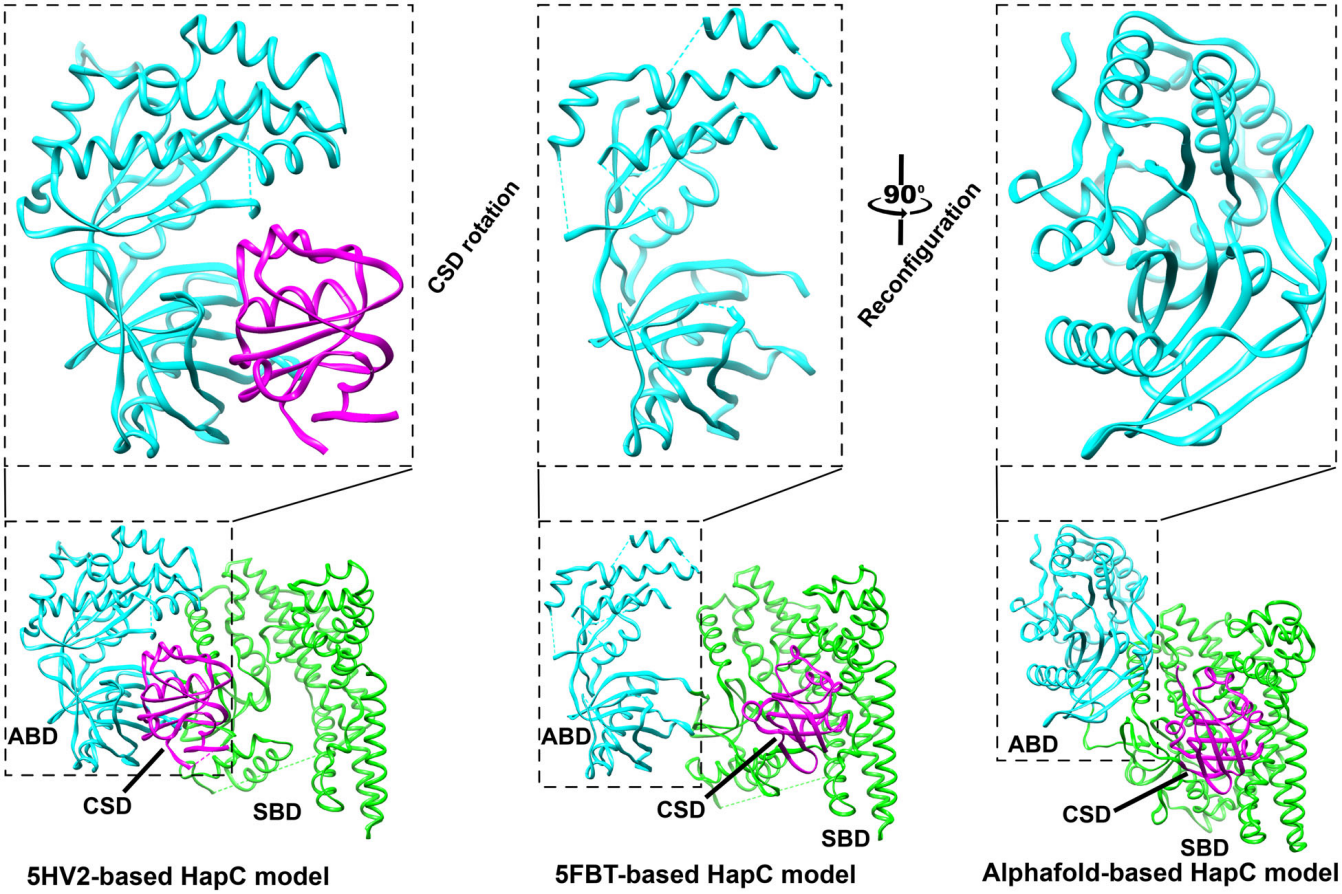
Comparison of the ABD and CSD orientations among the HapC models derived from PDB 5HV2 (left) and 5FBT (middle) and the AlphaFold‐based HapC model (right). The dashed lines represent unresolved regions in the template structure. The ABDs exhibit a substantial reconfiguration of approximately 90° between the 5FBT‐based models and the AlphaFold‐based model. ABD (cyan); SBD (green); CSD (magenta).

Structural analysis of the 5HV1‐based HapC model revealed a three‐domain architecture consisting of the ABD, CSD, and the SBD. In the PDB 5HV1 template structure, RPH harbors ligands bound to both the ABD and SBD, forming a well‐resolved complex that offers critical structural references. However, simultaneous ligand binding to both the ABD and SBD domains of RPH is less favorable under physiological conditions [35]. To better reflect the functionally relevant conformation, we examined alternative structural states of the same RPH. A HapC model based on PDB 5FBT was constructed, featuring a ligand bound to the SBD while the ABD remains unoccupied, with the CSD positioned near the SBD [35]. Another HapC model, derived from an apo form structure PDB 5HV2 [43], shows the ABD with the CSD in close proximity, representing an alternative conformation (Fig. 5). These RPH‐based templates support modeling of HapC in multiple conformations, demonstrating the coordinated dynamics of its three domains.

In recent years, AlphaFold has become a powerful tool for predicting protein structures. For comparison, an AlphaFold‐predicted HapC structure (UniProt: Q2S9J9) [44, 45] was also analyzed. Similar to the RPH‐based model, the AlphaFold‐predicted structure displays a confident three‐domain architecture (average pLDDT ≈ 90). However, the ABD domain is rotated 90° compared to the RPH‐based model (Fig. 5), possibly due to missing crystallization constraints or intrinsic flexibility driven by the I313–N332 loop connecting the ABD and SBD. Additionally, the AlphaFold‐predicted homolog most structurally similar to HapC is phosphoenolpyruvate synthase (PEPS) (AF‐A0A0F2J9F7‐F1‐v4) [45]. PEPS belongs to the pyruvate phosphate dikinase (PPDK)/PEPS superfamily and, similar to RPH, utilizes a catalytic histidine within a swiveling domain to mediate phosphate transfer [35, 46]. However, sequence identity among HapC, PEPS, and RPH is low, with all pairwise identities below 30%, suggesting functional conservation through evolution, rather than sequence similarity, accounts for the observed structural resemblance.

### Three phases of the enzymatic reaction in HapC

**Fig. 6:**
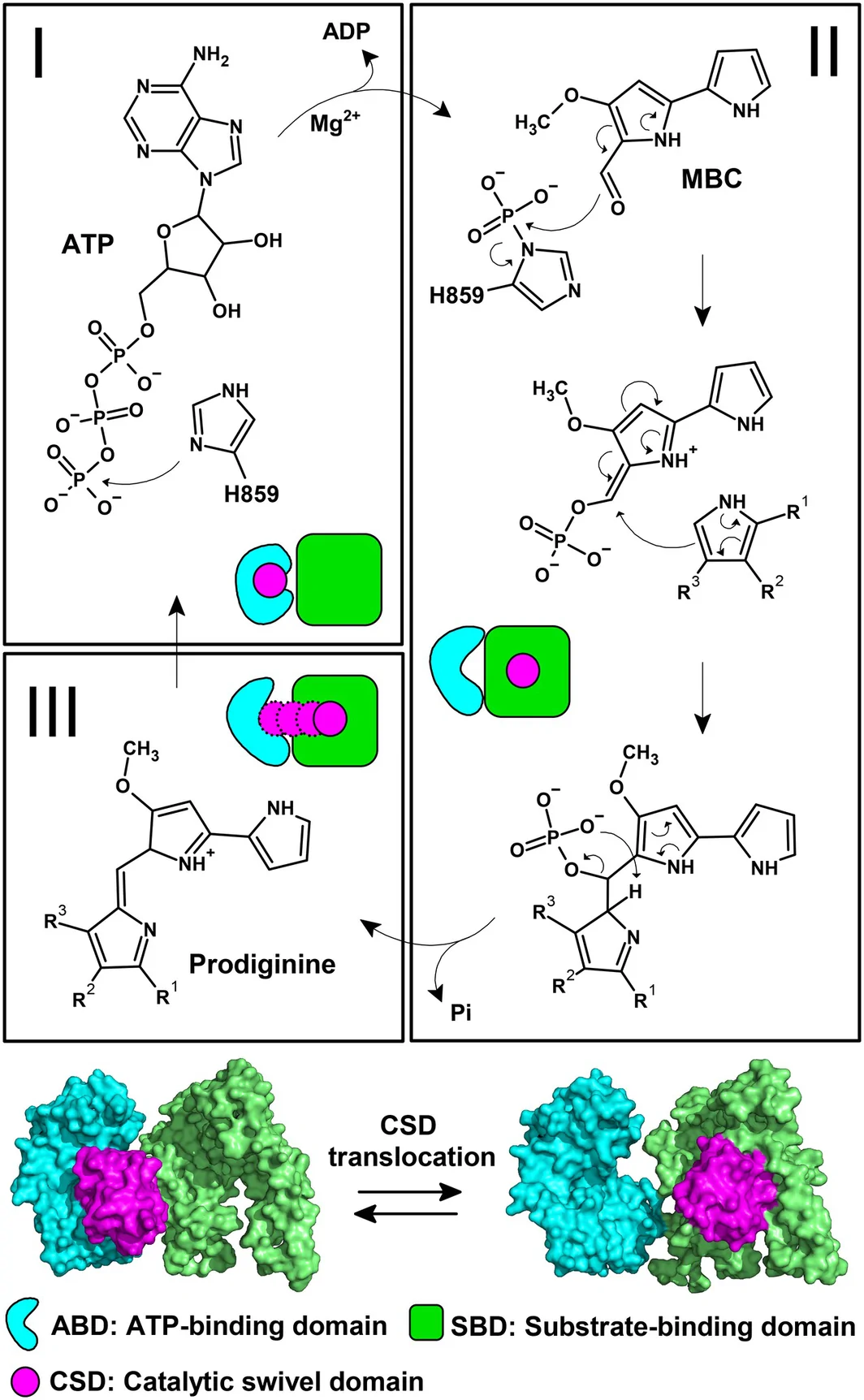
Proposed enzyme reaction mechanism of HapC. The curved arrows indicate the movement of electrons. The HapC model includes three structural domains: ABD (cyan), SBD (green), and CSD (magenta). Phase I: the ABD adopts a closed ATP‐grasping conformation, and the CSD is near the ABD. Phase II: the ABD adopts an open conformation, while the CSD swings toward the SBD. Phosphorylation of MBC is facilitated via H859. Phase III: the prodiginine is released, and the majority of the CSD population remains bound to the SBD (shown with solid line). A subset of the CSD exists in a transient state (shown with dashed lines), transitioning between the SBD and ABD. The bottom panel shows surface representations of two HapC models. The left model, based on PDB 5HV2, shows the CSD (magenta) positioned near the ABD (cyan). In contrast, the right model, derived from PDB 5HV1, shows the CSD rotated toward the SBD (green).

Structural comparisons based on crystallographic references and simulations reveal that the catalytic activity of HapC depends on coordinated domain interplay. Building on these insights, we propose a more explicit reaction mechanism for HapC. The catalytic cycle is delineated into three distinct phases, each defined by the dynamic positioning of the ABD, SBD, and CSD domains (Fig. 6).

Phase I, referred to as the activation phase, is characterized by ATP binding to the ABD, with the CSD positioned proximal to the ABD (Table 2; Fig. 6), as inferred from the 5HV2‐based HapC model (Fig. 5) and the behavior of RPH [35, 43]. The catalytic activity of HapC is dependent on both ATP and Mg^2+^. This interaction facilitates the transfer of the γ‐phosphate from ATP to H859, resulting in the formation of phosphorylated H859 (H859‐P) and the release of ADP [9]. The key residues involved in this process are highly conserved between HapC and RPH (Fig. S11). During this phase, the ABD undergoes a conformational change. Like RPH and PPDK [35, 47, 48], HapC may adopt a ‘closed’ ATP‐grasping conformation to facilitate ATP binding (Fig. 6).

**Table 2:** Proposed conformational states of HapC during the reaction cycle. The states are categorized into Phase I, Phase II, and Phase III. Each phase corresponds to a distinct functional state, accompanied by a specific pattern of domain coordination. The solid circles (●) denote the dominant conformations, whereas the hollow circles (**○**) represent the less prevalent conformations. Abbreviations: ABD engaged, the CSD interacts with the ABD; SBD engaged, the CSD interacts with the SBD; Transient, a dynamic conformation transition between ABD and SBD engagements.

Reaction phase	Phase I	Phase II	Phase III
Functional state	Enzyme activation	Substrate‐engaged	Product‐release
ABD engaged	●	**○**	
SBD engaged		●	●
Transient			**○**

Phase II, designated as the condensation phase, begins with the binding of the MBC in the SBD. The proportion of the CSD in the SBD‐engaged state increases [35] (Table 2). H859‐P then transfers its phosphate group to MBC, accompanied by L857, both derived from the CSD, while R696 in the SBD stabilizes the intermediate through hydrogen bonding (Fig. 4B). The nitrogen atom of the incoming MAP analog then attacks the MBC‐P, resulting in covalent bond formation and the generation of a coupled intermediate [49] (Fig. 6).

Following the dissociation of phosphate (Pi), the reaction enters Phase III, corresponding to the prodiginine release state and absence of ligands. During this phase, the majority of the CSD population remains associated with the SBD as its ground‐state [35], while a subset dynamically transitions between the SBD‐ and ABD‐engaged states (Table 2) (Fig. 6). This behavior preserves the readiness required to initiate a new catalytic cycle. The system resets to Phase I when a new ATP molecule binds to the ABD. Dynamic coordination of HapC domains underlies prodiginine biosynthesis.

In summary, this study integrates structural modeling and functional assays to define the mechanistic basis of HapC. Using short‐chain MAP analogs with varied alkyl positions, we show that HapC accommodates a range of pyrrole substrates. Docking simulations clarified ligand‐binding modes across catalytic stages and identified key active‐site residues. Together with domain‐level comparisons to homologous enzymes, these findings support a reaction mechanism involving dynamic coordination in HapC. This work advances understanding of prodiginine biosynthesis and may inform future pharmaceutical applications.

## Materials and methods

### Protein sequence analysis and structural modeling

The protein selected for this study was prodigiosin synthetase (HapC) from *H. chejuensis* KCTC 2396. The HapC sequence (WP_011399733.1) was retrieved from the NCBI database. Suitable template structures were identified using PDBsum [50]. The target templates were aligned using ClustalW. Boxshade formatting was achieved using the online tool available at Junli's website (junli.netlify.app/apps/boxshade) [51, 52]. The homologous structure with the highest alignment score was selected as the template. The three‐dimensional structure of HapC was predicted using the SWISS‐MODEL server [53]. The amino acid sequence of HapC was submitted to the server, which identified the structure of rifampin phosphotransferase (RPH, PDB ID: 5HV1, 5HV2, 5FBT) [35, 43] as the template. The resulting homology model of HapC was analyzed using UCSF Chimera [54, 55]. Protein structural information for the AlphaFold‐predicted structures was obtained from UniProt (accession IDs: Q2S9J9 (HapC) and Q5W252 (PigC)) via the AlphaFold database [44, 45].

### Cloning, expression, and purification of HapC

The *hapC* gene, encoding the 908‐residue condensation enzyme from *H. chejuensis* KCTC 2396, was chemically synthesized. The *hapC* gene sequence followed by a glycine‐serine linker, a thrombin cleavage site, and a C‐terminal 8 × His‐tag was subsequently cloned and inserted into the pET‐21a(+) vector (Novagen) using the *Nde*I and *BamH*I restriction sites. The resulting plasmid was transformed into *Escherichia coli* BL21(DE3), and the cells were subsequently grown in Luria–Bertani medium supplemented with 100 μg·mL^−1^ ampicillin at 37 °C. When the optical density (OD_600_) reached 0.6, protein expression was induced with 0.1 mm isopropyl *β*‐D‐1‐thiogalactopyranoside (IPTG), followed by incubation overnight at 16 °C. The cells were harvested by centrifugation, resuspended in Tris buffer (50 mm Tris/HCl and 500 mm NaCl; pH 8.0) and disrupted using a Constant Systems homogenizer (Labindia Instruments). The clarified lysate was obtained by centrifugation (48 000 × g, 30 min, 4 °C) and filtered through a 0.22 μm membrane. The obtained crude protein extract was used for subsequent *in vitro* activity assays.

To confirm the expression of HapC, the protein was purified and analyzed by the following procedure: The crude protein extract was subjected to affinity chromatography using a HisTrap™ HP column (Cytiva) and eluted with imidazole buffer (50 mm Tris/HCl, 200 mm imidazole, and 500 mm NaCl; pH 8.0). The purified HapC protein was buffer‐exchanged into Tris buffer (50 mm Tris/HCl and 150 mm NaCl; pH 8.0) and concentrated using an Amicon^®^ Ultra Centrifugal Filter (Millipore). SDS–polyacrylamide gel electrophoresis (SDS/PAGE) was conducted to confirm that the molecular weight of the purified HapC corresponded to the predicted size (Fig. S13).

### Enzymatic assays and mass verification

An *in vitro* assay for prodiginine biosynthesis using crude protein extract was conducted following a modified protocol from Chawrai *et al*. [9]. The 5 mL reaction mixture consisted of 5 mm Tris/HCl (pH 8.0), 100 mm NaCl, 1.2 mm MgCl_2_, 0.22 mm MBC (1), 0.22 mm monopyrrole (2a–2g, Table 1), 0.74 mm adenosine triphosphate (ATP), and 20% dimethyl sulfoxide (DMSO). The reaction was initiated with 3 mg of crude protein extract, while a reaction mixture without the extract served as a negative control. Incubation was performed at 25 °C for 16 h on a shaker at 100 rpm. After incubation, the reaction solutions were directly analyzed with a Beckman DU530 UV–Vis spectrophotometer to obtain full‐spectrum wavelengths and identify the absorption peaks of the products, followed by molecular weight analysis using liquid chromatography–mass spectrometry (LC–MS).

The analysis was performed using a Bruker Maxis Impact series LC–MS system equipped with a diode array detector and an ESI electrospray mass detector. Substances were separated using a reversed‐phase stationary phase BEH C18 column (1.8 μm, 2.10 × 100.00 mm). Chromatographic separation was performed using a gradient elution program. The mobile phase consisted of water containing 0.1% (v/v) formic acid (solvent A) and methanol containing 0.1% (v/v) formic acid (solvent B). Prior to running the gradient program, the column was equilibrated at initial conditions (95 : 5, A : B) for 5 min. The gradient program was set as follows: 0.00–2.00 min, 95 : 5 (A : B); 2.00–6.00 min, linear gradient to 100% B; 6.00–10.00 min, 100% B; 10.00–11.00 min, linear gradient to 95 : 5; and 11.00–15.00 min, 95 : 5. The flow rate was set to 0.4 mL·min^−1^, and the column temperature was maintained at 30 °C, with a sample volume of 1 μL injected per run. The mass detection mode was set to positive ion mode, with a scan range of *m/z* 50–1500.

### Molecular structure determination by NMR

The reaction products were purified prior to nuclear magnetic resonance (NMR) analysis. Prodiginines were extracted using ethyl acetate (EA) after incubation. Following the removal of EA via a rotary evaporator under reduced pressure, an appropriate amount of methanol solution containing 4% DMSO was added. Purified prodiginines were obtained via medium‐pressure liquid chromatography (MPLC) using a C18 column (RediSep Gold, 50 HP C18) with a linear methanol–water gradient containing 0.05% formic acid. The elution was monitored at 535 nm by absorbance measurement, and purified fractions were subsequently analyzed by NMR.

NMR data were acquired in Shigemi tubes with 100% (v/v) *D*_*2*_‐DMSO and *D*‐chloroform. All NMR experiments were carried out at 25 °C on Bruker Avance 800 MHz, 600 MHz, or 500 MHz NMR spectrometers equipped with a 5 mm triple‐resonance cryoprobe and a single‐axis pulsed field gradient by using standard pulse programs made available by the manufacturer. The data were acquired and processed using XwinNMR, version 3.5 (Bruker, Germany).

### Computational ligand docking and visualization

Ligand docking studies were conducted to determine the substrate‐binding residues in HapC. Molecular docking was performed using the CDOCKER module in Discovery Studio [56]. Compounds were first prepared using the Ligand Preparation module to generate 3D coordinates and assign appropriate protonation states. To define the binding sites, the HapC structure was aligned with the crystal structure of 5HV1. The ATP‐binding and substrate‐binding sites were identified based on the positions of the cocrystallized ligands ANP and RFP in 5HV1, respectively. The HapC structure was further prepared using the Protein Preparation module. Compounds were then docked into the respective binding sites using the CDOCKER module with default parameters, and the resulting poses were analyzed to evaluate molecular interactions between the compounds and binding site residues. To examine the interactions within the SBD, 3D structures of MBC‐P and the product 3g were similarly docked into the SBD binding pocket. To compare the structural and functional characteristics of HapC, the HapC structure was superimposed onto RPH. The resulting structures were visualized and analyzed using UCSF Chimera [55].

## Author contributions

YTC and ADR performed experiments, analyzed data, and contributed to manuscript preparation. HJH designed and conducted experiments. SFH and TEL performed experiments. WJH and KCH analyzed data, with KCH also contributing to manuscript preparation. SN contributed to manuscript preparation. PC designed experiments and provided reagents. AHJW and CCL supervised the study and contributed to manuscript preparation.

## Conflict of interest

The authors declare no competing interests.

## Supporting information


**Fig. S1.** Gene cluster for the biosynthesis of prodigiosin.
**Fig. S2.** Model evaluation of HapC: Ramachandran plot and stereochemical validation.
**Fig. S3.** Extracted ion chromatograms of enzymatic products 3a–3g generated by HapC.
**Fig. S4.** NMR spectrum of 2‐methyl‐3‐pentyl‐6‐methoxyprodiginine (3a, prodigiosin).
**Fig. S5.** NMR spectrum of 2‐methyl‐6‐methoxyprodiginine (3b).
**Fig. S6.** NMR spectrum of 2,3‐dimethyl‐6‐methoxyprodiginine (3c).
**Fig. S7.** NMR spectrum of 2,4‐dimethyl‐6‐methoxyprodiginine (3d).
**Fig. S8.** NMR spectrum of 3,4‐dimethyl‐6‐methoxyprodiginine (3e).
**Fig. S9.** NMR spectrum of 2‐ethyl‐6‐methoxyprodiginine (3f).
**Fig. S10.** NMR spectrum of 3‐ethyl‐2,4‐dimethyl‐6‐methoxyprodiginine (3g).
**Fig. S11.** Superimposition of the ATP‐binding domain of the HapC model and rifampin phosphotransferase (PDB ID: 5HV1).
**Fig. S12.** Sequence alignment of HapC, rifampin phosphotransferase, and PigC.
**Fig. S13.** SDS–PAGE analysis of HapC.
**Data S1.** NMR analysis of compounds synthesized by HapC (including Figs S4–S10).

## Data Availability

All data supporting the findings of this study are available within the article (see Figs 1, 2, 3, 4, 5, 6 and Tables 1, 2) and its Supporting Information.
