# Supplementary material for: Mechanistic insights into HapC: A key enzyme in prodiginine biosynthesis in Hahella chejuensis
Source: FEBS J. 2026 Mar 30;293(17):5303–17. doi: 10.1111/febs.70518 (PMC13534948; doi:10.1111/febs.70518)
Supplement: Supplementary file 1 [file FEBS-293-5303-s001.docx]

**Supporting Information**

**Mechanistic Insights into HapC: A Key Enzyme in Prodiginine Biosynthesis in *Hahella chejuensis***

Yueh-Te Chan^1#^, Aden Dhana Rizkita^2#^, Hsin-Ju Hsieh^3,4^, Shu‐Fang Hsu^1^, Tony Eight Lin^5,6^, Wei-Jan Huang^2,7^, Kai-Cheng Hsu^5,6,7^, Sheila Nathan^8^, Peiching Chang^4^, Andrew H.-J. Wang^1*^ and Cheng-Chung Lee^1,9*^

^1^The Ph.D. Program for Translational Medicine, College of Medical Science and Technology, Taipei Medical University, Taipei 110, Taiwan.

^2^School of Pharmacy, College of Pharmacy, Taipei Medical University, Taiwan.

^3^Department of Water System Development and Application Research, Division of Water Technology, Industrial Technology Research Institute, Hsinchu 310, Taiwan.

^4^Material and Chemical Research Laboratories, Industrial Technology Research Institute, Hsinchu 310, Taiwan.

^5^Graduate Institute of Cancer Biology and Drug Discovery, College of Medical Science and Technology, Taipei Medical University, Taipei, Taiwan.

^6^Ph.D. Program for Cancer Molecular Biology and Drug Discovery, College of Medical Science and Technology, Taipei Medical University, Taipei, Taiwan.

^7^Ph.D. Program in Drug Discovery and Development Industry, College of Pharmacy, Taipei Medical University, Taipei, Taiwan.

^8^Department of Biological Sciences and Biotechnology, Faculty of Science and Technology, Universiti Kebangsaan Malaysia, Selangor, Malaysia.

^9^International Ph.D. Program for Translational Science, College of Medical Science and Technology, Taipei Medical University, Taiwan.

^#^ These authors contributed equally to this work.

*Correspondence: [ahjwangsync@gmail.com](mailto:ahjwangsync@gmail.com), [chengung@tmu.edu.tw](mailto:chengung@tmu.edu.tw)

Figure S1. The gene cluster for the biosynthesis of prodigiosin. Genes are denoted by arrows. The yellow arrows are genes encoding condensation enzymes (HapC, PigC); the green arrows represent genes for the biosynthesis of the monopyrrole (MAP); the open arrows are genes required for the biosynthesis of 4-methoxy-2,2'-bipyrrole-5-carboxyaldehyde (MBC); and the gray arrows are flanking genes or genes with unknown functions.

**Figure S2.** **Model evaluation of HapC. Ramachandran plot and stereochemical validation. The homology model of HapC was analyzed. The Ramachandran plot shows 93.63% of residues in favored and allowed regions, with a clash score of 2.34 and a MolProbity score of 1.98. Model validation was performed using the SWISS-MODEL structure assessment tools.**

**Figure S3.** Extracted ion chromatograms (EICs) of enzymatic products 3a–3g generated by HapC are shown in Panels A–G, respectively (see Table 1 for the corresponding analog structures). Panel H shows the blank injection (EIC at m/z 296, corresponding to product 3g), confirming the absence of residual signal. Consistent results were observed for all products 3a–3g. These chromatograms were generated based on the calculated [M+H]⁺ values of each compound. The x-axis represents the retention time, and the y-axis indicates the relative ion intensity (Intens.). The corresponding high-resolution mass spectra are shown in the main text (Figure 3, panels A–G). In panels D and E, minor peaks are observed at earlier retention times (RT ≈ 0.6 min). These peaks share a fragment ion at m/z 239.10, consistent with in-source fragmentation involving loss of an ~29 Da neutral species (best match CHO, 29.0027 Da).

**Supplementary Text S1.** NMR Analysis of Compounds Synthesized by HapC.

The names are in accordance with the IUPAC nomenclature.

**2-Methyl-3-pentyl-6-methoxyprodiginine (3a, prodigiosin)**

1H NMR (600 MHz, CDCl_3_) δ 13.36 (br.s, 1H), 12.73 (br.s, 1H), 12.56 (br.s, 1H), 7.26 (s, 1H), 6.94 (s, 1H), 6.95–6.91 (m, 1H), 6.67 (s, 1H), 6.36 (dt, J = 4.0, 2.0 Hz, 1H), 6.08 (s, 1H), 4.00 (s, 3H), 2.45 (s, 3H), 2.39 (t, J = 7.6 Hz, 2H), 1.54 (t, J = 7.4 Hz, 2H), 1.38–1.31 (m, 2H), 1.32 (dd, J = 4.8, 2.7 Hz, 2H), 0.90 (t, J = 6.9 Hz, 3H).

Figure S4. NMR spectrum of 2-methyl-3-pentyl-6-methoxyprodiginine (3a, prodigiosin).

**2-Methyl-6-methoxyprodiginine (3b)**

1H NMR (600 MHz, CDCl_3_) δ 7.27 (s, 1H), 7.02 (s, 1H), 6.97 (ddd, J = 3.9, 2.5, 1.3 Hz, 1H), 6.84 (t, J = 3.2 Hz, 1H), 6.38 (dt, J = 4.1, 2.3 Hz, 1H), 6.18 (dd, J = 4.1, 1.8 Hz, 1H), 6.10 (d, J = 1.9 Hz, 1H), 4.03 (s, 3H), 2.62 (s, 3H).

Figure S5. NMR spectrum of 2-methyl-6-methoxyprodiginine (3b).

**2,3-Dimethyl-6-methoxyprodiginine (3c)**

1H NMR (600 MHz, CDCl_3_) δ 7.23 (s, 1H), 6.94 (s, 1H), 6.92 (t, J = 3.0 Hz, 1H), 6.66 (s, 1H), 6.36 (p, J = 2.5, 1.8 Hz, 1H), 6.08 (s, 1H), 4.01 (s, 3H), 2.54 (s, 3H), 2.04 (s, 3H).

Figure S6. NMR spectrum of 2,3-dimethyl-6-methoxyprodiginine (3c).

**2,4-Dimethyl-6-methoxyprodiginine (3d)**

1H NMR (600 MHz, DMSO) δ 7.08 (q, J = 2.0 Hz, 1H), 6.70 (dt, J = 3.4, 1.6 Hz, 1H), 6.63 (s, 1H), 6.21 (dt, J = 3.6, 1.9 Hz, 1H), 6.12 (s, 1H), 5.84 (t, J = 0.8 Hz, 1H), 3.85 (s, 3H), 2.38 (s, 3H), 2.13 (s, 3H).

Figure S7. NMR spectrum of 2,4-dimethyl-6-methoxyprodiginine (3d).

**3,4-Dimethyl-6-methoxyprodiginine (3e)**

1H NMR (500 MHz, CDCl_3_) δ 13.32 (s, 1H), 7.28 (s, 1H), 7.27 (s, 1H), 6.97–6.92 (m, 1H), 6.36 (t, J = 3.1 Hz, 1H), 6.07 (s, 1H), 4.02 (s, 3H), 2.21 (s, 3H), 2.04 (s, 3H).

Figure S8. NMR spectrum of 3,4-dimethyl-6-methoxyprodiginine (3e).

**2-Ethyl-6-methoxyprodiginine (3f)**

1H NMR (500 MHz, DMSO) δ 7.07 (t, J = 2.3 Hz, 1H), 6.72 (d, J = 3.6 Hz, 1H), 6.64 (s, 1H), 6.57 (d, J = 3.7 Hz, 1H), 6.21 (t, J = 3.2 Hz, 1H), 6.12 (s, 1H), 6.00 (d, J = 3.7 Hz, 1H), 3.83 (s, 3H), 2.76 (q, J = 7.6 Hz, 2H), 1.23 (t, J = 7.3 Hz, 3H).

Figure S9. NMR spectrum of 2-ethyl-6-methoxyprodiginine (3f).

**3-Ethyl-2,4-dimethyl-6-methoxyprodiginine (3g)**

1H NMR (600 MHz, CDCl_3_) δ 7.22 – 7.19 (m, 1H), 7.05 (s, 1H), 6.88 (p, J = 1.6 Hz, 1H), 6.34 (dt, J = 4.2, 2.3 Hz, 1H), 6.09 (d, J = 2.0 Hz, 1H), 4.01 (s, 3H), 2.55–2.23 (m, 3H), 1.07 (t, J = 7.6 Hz, 3H).

Figure S10. NMR spectrum of 3-ethyl-2,4-dimethyl-6-methoxyprodiginine (3g).

**Figure S11.** Superimposition of the ATP-binding domain (ABD) of the HapC model and rifampin phosphotransferase (PDB ID: 5HV1). The ATP molecule from HapC and the AMPPNP molecule from the 5HV1 structure are shown as ball-and-stick representations colored yellow and magenta, respectively. **Conserved residues are depicted as cyan (HapC) and orange (5HV1) lines, and Mg²⁺ ions as light green (HapC) and dark gray (5HV1) spheres.** The HapC homology model was generated using the SWISS-MODEL server, and structural superimposition and visualization were performed using UCSF Chimera.

Figure S12. Sequence alignment of HapC (UniProt ID: Q2S9J9), rifampin phosphotransferase (PDB ID: 5HV1), and PigC (UniProt ID: Q5W252). Conserved residues are shaded in black. Ligand-binding residues within the ATP-binding domain (ABD) and the substrate-binding domain (SBD) are marked in blue and green, respectively. The catalytic residues in CSD are highlighted in magenta. Sequence alignment was performed using ClustalW, and formatted using Boxshade.

Figure S13. SDS‒PAGE analysis of HapC. M: Molecular weight marker; P: pellet fraction; T: total cell lysate (crude extract); FL: flow-through from the Ni-NTA affinity column; W: wash fraction. The molecular weight (MW) of purified HapC was approximately consistent with the predicted size of 104 kDa.
